# Medicinal plants used in the management of cancers by residents in the Elgon Sub-Region, Uganda

**DOI:** 10.1186/s12906-023-04273-5

**Published:** 2023-12-12

**Authors:** Ali Kudamba, Josephine N. Kasolo, Godfrey S. Bbosa, Allan Lugaajju, Henry Wabinga, Nixon Niyonzima, Moses Ocan, Ali M. Damani, Hussein M. Kafeero, Jamilu E. Ssenku, Shaban O. Alemu, Muhammad Lubowa, Abdul Walusansa, Haruna Muwonge

**Affiliations:** 1 Department of Physiology, School of Biomedical Sciences, College of Health Sciences, Makerere University, Kampala, Uganda; 2 Faculty of Health Sciences, Habib Medical School, Islamic University in Uganda, Kampala, Uganda; 3 Faculty of Science, Department of Biological Sciences, Islamic University in Uganda, Mbale, Uganda; 4 Department of Pathology, School of Biomedical Sciences, College of Health Sciences, Makerere University, Kampala, Uganda; 5 Department of Pharmacology & Therapeutics, School of Biomedical Sciences, College of Health Sciences, Makerere University, Kampala, Uganda; 6 Uganda Cancer Institute, Kampala, Uganda; 7 Faculty of Science, Department of Food Science & Nutrition, Islamic University in Uganda, Mbale, Uganda

**Keywords:** Cancer, Ethnobotanical survey, Herbalists, Elgon sub-region, Medicinal plants

## Abstract

**Background:**

In Uganda, medicinal plants have been utilized to treat a variety of ailments, including cancer. However, there is little information available about the medicinal plants used to treat cancer in the Elgon subregion. As a result, the current study documented the plant species used in the management of cancer in the Elgon sub-region.

**Methods:**

Data were gathered by observation, self-administered questionnaires, interview guides, and guided field trips. Analyzing descriptive statistics and creating graphs were done using SPSS (version 21.0) and GraphPad Prism® version 9.0.0, respectively. Well-established formulae were used to calculate quantitative indices. The narratives were interpreted using major theories and hypotheses in ethnobotany.

**Results:**

A total of 50 plant species from 36 families were documented, and herbal knowledge was mainly acquired through inheritance. Fabaceae and Asteraceae comprised more plant species used in herbal preparation. Most plants were collected from forest reserves (63%); herbal therapies were made from herbs (45%); and leaves were primarily decocted (43%). The most frequently used plants were *Tylosema fassoglensis*, *Hydnora abyssinica*, *Azidarachata indica*, *Prunus Africana*, *Kigelia africana*, Syzygium *cumini*, *Hydnora africana, Rhoicissus tridentata*, *Albizia coriaria, and* *Plectranthus cuanneus*. All the most commonly used plants exhibited a high preference ranking (60–86%) and reliability level (74.1–93.9%). Generally, the ICF for all the cancers treated by medicinal plants was close to 1 (0.84–0.95).

**Conclusions:**

The ten most commonly utilized plants were favored, dependable, and most important for treating all known cancers. As a result, more investigation is required to determine their phytochemistry, toxicity, and effectiveness in both in vivo and in vitro studies. This could be a cornerstone for the pharmaceutical sector to develop new anticancer medications.

**Supplementary Information:**

The online version contains supplementary material available at 10.1186/s12906-023-04273-5.

## Introduction

Cancer is the world's most serious public health problem [1]. Over the previous decades, it was responsible for 18.1 million cases and almost 10 million deaths [1]. Cancer cases will more than double within the next two decades, reaching 43.5 million as per GLOBOCAN [2]. According to the WHO, cancer is the first or second leading cause of deaths before the age of 70 years in 112 countries and third or fourth in 23 countries [2]. The African Cancer Statistics, highlighted that Africa has the highest cancer mortality rate but the lowest incidence rate [3]. This is due to late diagnosis, exorbitant treatment costs, and, most critically, the difficulty in accessing conventional drugs [3]. Antitumor drug resistance in cancer cells contributes to some mortality [3], and given these shortcomings, the quest to search for medicinal plants as alternative remedies, including the use of medicinal plants, remains among the viable options. In 2020, the Global Cancer Observatory recorded 1,414,259 new cases of prostate cancer and 375,305 deaths [4]. Prostate cancer cases increased from 10,200 in 1990 to 21,900 in 2010, with fatalities ranging from 5600 to 12,300 during the same period [4]. Prostate cancer cases increased from 10,200 in 1990 to 21,900 in 2010, with fatalities ranging from 5600 to 12,300 during the same period [5]. In 2020, a total of 77,300 cases were reported in Sub-Saharan Africa [6]. A estimate of 1329 deaths from prostate cancer-related causes and over 2375 new cases were reported in Uganda in 2017 [7]. In Uganda, the prevalence of prostate cancer was estimated to be 6.4% in 2018, and by 2030, it is expected to reach 33.4% [8]. The possibility of underestimation of cancer cases cannot be ruled out because Uganda has inadequate cancer diagnostic resources [7, 9]. Despite the lack of well-documented cancer cases in the Elgon sub-region, health professionals interviewed at Mbale Regional Referral Hospital (MRRH) revealed an annual rise in disease. The lack of a cancer registry and the remoteness of the present study area are likely to make the situation more fragile than what is documented in Kampala, the capital city of Uganda.

On a global scale, there are numerous cancer treatment options, including chemotherapy, radiotherapy, surgery, hormone therapy, immunotherapy, photodynamic therapy, targeted therapy, gene therapy, and stem or bone marrow transplants [10]. In developing countries, particularly in Africa, where Uganda lies, the accessibility and affordability of diagnostic and conventional cancer treatment are limited by the inadequate or lack of specialized clinics in the country [10]. The use of conventional drugs has been tainted by serious side effects because of their lack of specificity, characterized by the death of both cancer and healthy cells, which even exacerbates the fragile situation [10]. Additionally, there have been cases of secondary cancers developing after treatment, and current drugs are ineffective for treating advanced disease stages. Therefore, the use of plant extracts cannot be entirely disregarded across all global populations, including the Elgon sub-region.

Approximately 91% of the licensed and approved cancer medicines come from medicinal plants, making them important sources of conventional cancer medications like paclitaxel (Taxol), vincristine (Oncovin), vinorelbine (Navelbine), and teniposide (Vumon) [10, 11]. In Sub-Saharan Africa, more than 80% of the population, particularly in developing countries where Uganda lies, relies on herbs directly to meet their primary healthcare needs [12–14]. However, the use of medicinal plant species may vary across the different regions of Uganda due to variations in the ecological setup and cultural variability. For example, *Moringa oleifera*, Lam, *Vernonia amygdalina* Dell, *Warburgidia ugandenesis* Sprague, *Carica papaya* L, *Annona muricata*, and *Biden pilosa*, among others, were largely reported to be used in the management of cancers in Central and Western Uganda [15–17]. In the Elgon sub-region, there is, however, little accessible information in this regard. Therefore, it is possible that the plant species used to treat cancers in the Elgon sub-region are distinct from those employed in other parts of the country, which necessitates urgent investigation.

Uganda is home to more than 6,000 different plant species with medicinal benefits [14, 15]. Medicinal plants have become more important in the ethnobotanical approach to treating a range of illnesses, including cancer [14, 15]. In Uganda, there are more than 3000 anticancer medicinal plants, containing over 5000 phytochemicals such as phenolics, carotenoids, glucosinolates, terpenoids, and alkaloids, known to form integral components in cancer treatment [18]. The Elgon sub-region is one of the areas in the world that is blessed with a variety of medicinal plant species, and the possibility that some have cancer treatment benefits cannot be ruled out [19]. Accurate documentation of traditional knowledge has always been critical for preservation and has served as a foundation for plant-based medication development [13]. However, indigenous knowledge of the plants used to treat cancer has not been extensively documented, despite the growing assumption of their use in this region [20]. Therefore, an ethnobotanical survey was conducted in the Elgon sub-region with the aim of identifying plant species used in the management of cancer.

## Materials and methods

### Study area

The study was conducted in two rural districts of Sironko and Bulambuli districts in the Elgon sub-region of eastern Uganda and are thought to have a range of plant species [19]. The distances between Sironko and Bulambuli districts and Mbale city and Kampala, the capital of Uganda are 24.7 km and 55.4 km, and 275.9 km and 306.8 km, respectively [21, 22]. Sironko district is bordered to the north by the Bulambuli district, the northeast by the Kapchorwa and Kween districts, the east by Kenya, the southeast by the Bududa district, and the southwest by the Mbale and Bukedea districts. To the north, east, and west of Bulambuli district are Nakapiripirit, Kapchorwa, and Bukedea districts respectively. The average elevation of these districts is 3996 feet (1,218 m) [21, 22]. According to [22], the average annual temperature is 24.4 oC, while there is between 920 and 1650 mm of rain per year on average [17]. The study was conducted in seven villages located at latitudes and longitudes indicated for each as Zema (1.212^o^E, 34.31^o^N), Suguta (1.24^o^N, 34.37^o^E), and Jewa (1.284^o^N, 34.31^o^E) in the Bulambuli district, Nakidoba (1.26° N, 34.49o E), Bulwala (1.25° N, 34.43° E), Miwu (1.21^o^N, 34.34° E), and Madaya (1.21^o^N, 34.37° E) in Sironko districts. The majority of the custodians of traditional herbal knowledge were elderly, ranging in age from 55 to 75, and they claimed to have been residents of this region for their entire lives. They have accumulated a broad range of ecological knowledge about the different medicinal plants, as shown by this, over time. However, because they are older than the recommended age range (31–45 years), this puts the sector's sustainability in danger, and grave concerns about collapse continue to be raised [12, 20]. As a result, an ethnobotanical study was carried out between September and November 2022 in seven villages, including Zema, Suguta, and Jewa in the Bulambuli district and Nakidoba, Bulwala, Miwu, and Madaya. The selection of these study sites was shaped under the guidance of local leaders and elders, who, to the best of their knowledge, believed they had the most experienced herbalists.

### Study design

The study used a mixed study design (both quantitative and qualitative). The investigators used semi-structured self-administered questionnaires, interview guides, guided field walks, and observations during the process of data collection. The questionnaires were used to collect data from all 45 herbalists (38 males and 7 females). In addition, a total of ten key informants, aged between 55 and 75, all male, were interviewed in Lumasaba, the native language of the area, using a pre-tested interview guide. GraphPad Prism® version 9.0.0 created descriptive statistics (frequency and percentage) and graphs, which were then displayed in a table and figure for simple comprehension.

### Demographic characteristics of the population

The majority of the population is made up of the Gisu, the seventh-largest tribe in Uganda, and Lumasaba speakers [23]. The community is characterized by being largely rural, with few peri-urban centers and a relatively high level of poverty and illiteracy [23]. This suggests that they are financially crippled to afford the costly cancer chemotherapy treatments, and therefore turning to alternative plant medicines is still an option.

### Participants’ selection, sample size, and sampling techniques

The sample size was attained when the next 10 participants in the data collection process were unable to elicit any fresh thoughts from those who had already been interviewed (redundancy criterion, saturation point) [24]. The researchers purposely chose local herbalists who had spent a lot of time there, indicating their familiarity with the region and their utilization of local resources, including plants, to meet their primary health care needs.

### Research instruments

Data were collected from herbalists through questionnaires, observation guides, and interview guides. Ten (10) herbalists who were available at the time of the study were surveyed to get information on the identification and use of local plant species. Translators with proficiency in Lumasaba, the local language, translated the interviews where necessary.

### Validity and reliability of research instrument

A reconnaissance visit was conducted for five days to gain a basic understanding of the potential villages suitable for our study. After the initial visit, two weeks were spent preparing research instruments, and another week was spent training research assistants on how to administer the instruments. A total of ten questionnaires and five interview guides were piloted. The result of the pilot was used to improve the efficiency of the research instrument for data collection.

To test the validity of the research instrument, a questionnaire was prepared and submitted to an ethnobotany researcher for cross-checking and to assess the reliability of the content. The reliability of the research instruments was tested during the pilot through the split-half technique and the Cronbach alpha coefficient [24]. Here, the instrument was provided to 10 herbalists, divided into two groups. The reliability of the items was based on estimates of the variability of responses between the two groups. In this study, the reliability coefficient was found to be 0.85, which was very good for analysis.

### Inclusion and exclusion criteria

Regardless of their level of experience, the study included all herbalists over the age of 18 who had a fundamental understanding of the plants used to treat cancer. Additionally, only herbalists who had lived in the area for over ten years had a basic grasp of the ecological framework of plants used in treating disease and were proficient in the native language [23, 25]. The study did not include any herbalists who did not match the aforementioned requirements.

### Ethnobotanical data collection, plant identification, and authentication

He self-administered questionnaire was used to collect data from all the herbalists who consented to participate in this study. In addition to this, a total of 10 key informants (senior herbalists) were interviewed to explore more about plants used in cancer management. All of their views and opinions were immediately recorded. Field-guided walks were also conducted, and during the process, observations of plant habits and photographs were taken with their consent. The collection of plant parts (shoots mainly and root tubers, where applicable) that were used for taxonomic classification. The plants were identified in situ by an expert using a plant-based field manual. The plant parts were wrapped in old newspapers and placed in the plant placement. The plant placement was transported to Makerere University's Herbarium Laboratory for further scrutiny on taxonomic classification. The vouchers were deposited therein for each of the identified medicinal plant species. The plant names were confirmed in the plant databases at IPNI (www.ipni.org) [26].

### Qualitative data analysis

Statistical Package for Social Scientists (SPSS, version 21) was used to analyze the data, and GraphPad Prism® (version 9.0.0) was used to create the graphs. Descriptive statistics (frequency and percentages) were utilized to examine data on plant parts used, plant habits, forms of preparation, and administration. The narrative analysis that was conducted on the participants' personal narratives was supported by the major hypotheses and ethnobotany theories. The pre-existing formulae were used to determine the fidelity level (FL), informant consensus factors (ICF), and preference rankings (PR). Quantitative Indices.

### Quantitative indices

#### Preference ranking

The preference ranking of ten medicinal plants for treating cancer was conducted after selecting ten key informants as earlier guided by [27], with modifications. For this case, 10 plant species that were commonly mentioned in relation to the treatment of cancer were chosen. The herbalists were given the responsibility of rating the plant above the cancers that it treats. Each informant was provided with the medicinal plants reported to treat cancer, either leaves or tubers, or both, which were paper-tagged, and then asked to assign the highest value (10) for most of the preferred species against the illness and the lowest value (1). The value given to each plant was summed up, and the rank for each species was determined based on the total score.

### Informants Consensus Factor (ICF)

The informant consensus factor checks how similar, agreed upon, or shared the information is when it comes to using plants to treat different types of cancer. This was calculated using the formula described by [28], with modifications. Low ICF indicates a lack of knowledge about the plants used, which leads to random plant selection among herbalists regarding their usage and disagreements over the species used to cure a specific ailment (in this case, cancer). ICF is high (quite close to 1), demonstrating that herbalists have a solid grasp of the species and a carefully designed criterion of communication among the community. As a result, it is assumed that a medicinal plant with a high informant consensus factor is effective and regularly used in this region to cure particular cancers*.* ICF was computed from the formula indicated below

$$ICF=\frac{Nur- Nt}{Nur-1}$$

where *N*_ur_ is total number of plant use reports (citations) for cancer category, and N_t_ is number of species used for treatment of that ailment or cancer category.

### Fidelity Level (FL) or level of trust

To determine the significance of these plants in treating cancer in this region, the fidelity of each of the top 10 plants was calculated. The fidelity level (FL) was calculated from the formula indicated below.

$$FL=\frac{Lp x 100\mathrm{ \%}}{Iu}$$

Where L_p_ represents the number of informants who suggested using a specific species for the same major use (prostate cancer), and l_u_ represents the total number of informants who mentioned using the same species for any other use.

## Results

The current study enrolled 45 participants, who were mostly male (84%), Ugandan (89%), and married (76%). More participants (33.3%) were in the age bracket of 55 to 75 years and were mostly peasants (76%). Furthermore, the vast majority of the participants had only completed primary school (78%) and were generally of low literacy level.

An interview with a senior herbalist and elder from Jewa and Zema villages revealed this information: "Culturally, in our villages, herbal practice by gender is taken as a responsibility for males, so females are rarely involved, except under special circumstances like maternity services and hygiene practices, and these are especially encouraged only for older women," said one of the senior herbalists from Nakidobo. "I got involved in herbal treatment when I was 10 years old; so I could not go far with formal education, and that is the major reason why I dropped out of primary three.”

## Source of knowledge of medicinal plants

According to the research, more herbalists (37%) claimed to have learned about medicinal plants from their grandparents and parents (inheritance), with spiritual direction and peer learning coming in second and third, respectively (22%), followed by dreams (12%) and very few cases of experimentation (6%). Nobody acquired knowledge of medicinal plants via technical education.

The herbalist's interview in this regard produced the following responses: "*My father and a grandfather, both of whom lived in a Jewa village, used to take me to a senior herbalist, who began showing me the" herbs he used to treat various maladies. According to Bulaba village's senior herbalist, I started treating individuals with certain ailments when I was 15 years old. An elderly herbalist who claimed to be 70 years old said, "I learnt to treat different diseases, including cancer, from my family, friends, and peers. Another experienced herbalist claimed that his grandfather taught him about herbs, and that his grandfather had learnt about some of the medicinal plants through dreams and divine direction.”*

### Medicinal plants, habits, cancer treated, and methods of preparation & route of administration

The herbalists disclosed a total of 50 plant species from 36 families that were suggested to have cancer treatment benefits. Plant families of Fabaceae (6) and Asteraceae (4), constituted the greater number of species used in cancer treatment. Herbs were more (45%), than all other plant habits and these were followed by trees (27%). More herbal therapies were prepared by decoction (40%) and was followed by concoction (26%) and were mainly administered orally.

### Preferred medicinal plant species

Interestingly, all the most frequently mentioned plants for cancer treatment scored a plant value above 50%. *Tylosema fassoglensis* (PV = 86) was rated as the most effective at treating all known malignancies in this area. The other plant species were more pronounced for the treatment of specific cancers.

### Informant Consensus Factor (ICF)

Generally, all the cancers treated by medicinal plants scored a higher ICF above 0.5 (0.84–0.95) and close to 1. The highest ICF score (0.95) was for intestinal cancer, while the lowest value was 0.84 for prostate cancer.

### Fidelity Level I (FL) of most preferred plants used plants in cancer

The findings revealed that, on average, herbalists trusted all 10 of the most prevalent plant species with fidelity indices above 50%, highlighting their significance to herbalists in cancer treatment. *Hydnora abyssinica* A. Br. received the highest score for treating prostate cancer (93.9%), while *Plectranthus cuanneus* had the lowest score (71.4%) for treating gastrointestinal cancer (71.4%).

### Source of medicinal plants

The majority of medicinal plants (63%) were collected from forest reserves, while the least (7%).

The following statements were made during an in-depth interview with key informants who were males between the ages of 51 and 60 from the villages of Suguta, Jews, and Bulwala*: "We usually use certain nearby plants from our homestead as prescribed by our grandparents; some plants are very effective at treating cancer, but honestly speaking, we collect most plants from far away, and some are in protected areas of the forest and cannot be easily accessed “Another elder from* Bulwala village *said that all my herbs for my cancer patient are plants that are readily available and accessible throughout the year"*

### Plant Parts used to prepare herbal remedies

The outcome suggests that, depending on the plant species and cancer type at hand, the utilization of plant parts differed substantially. In comparison to other plant parts, leaves (40%) and root bark (23%) were used to make more herbal remedies.

In response to an in-depth interview with a key informant, one of the elderly from Zema, Suguta, and Madaya villages stated, “I normally use leaves to prepare herbal therapies because they are readily available throughout the year, in addition to being most effective compared to other parts.” "I believe that some leaves have more medicinal properties than the stem and root barks”. "I uproot a small plant, boil it, and give it to my patients, who are cured after a while, depending on the type of disease."

### Plant habits

Results showed that herbs (45%) and trees (34%) comprised more herbal therapies (45%) for cancer treatment in this area.

Another local leader and elder in the villages of Nakidobo, Miwa Jewa, and Bulwala stated during an interview with a herbalist that “*I normally use small weeds as herbs in this area because they are abundant and available almost through the year and have also cured our patients after use." "However, some herbalists also stated that I also use some parts of the plant, such as stems, barks, and roots, despite the fact that some potential weeds are sometimes far from our homes."" In fact, according to the senior herbalist in Suguta village, some of the key plants are collected as far as 30 km from my home because they are very important*.

### Modes of preparation & route of administration of herbal therapies

The herbalists employ a range of herbal preparation methods and delivery systems. Decoction (40%) was more popular preparation method compared to all other methods, and oral administration route was more popular.

“We *normally use a mixture of plants and instruct our patients to drink them in the morning and at night because I believe that is the most effective means of treatment, and it can take half a nice cup for two to three months to cure." The herbalists in* Zema, Nakidobo, and Makogati said this during the interview. *"An herbalist from Jewa said, “In most cases, I don’t trust the use of a single plant to boil to treat a given disease; that is why I mix three or more, but every now and then, I can use one, which I believe is the strongest, for just a month." An elderly man among herbalists said "The treatment of patients with herbs is determined by the type of cancer I have’’. For all other cancers, I boil various plants and patients, but not skin cancer”, A local leader and senior herbalist advised me to burn the ash and apply it topically to the affected area while also drinking the same plants for three months*."

## Discussion

The study found that the majority of participants were men and Ugandans**.** The predominance of men is in line with African beliefs that men are more likely than women to operate as herbalists. It has also been noted that men predominate in the herbal industry in Butelejja and in rural Pakistani communities like Dhirkot and Azad Jammu and Kashmir [29, 30]. But prior studies in Indonesia, Iraq, and the Kurdistan Regions revealed that women controlled the herbal industry in those countries. These were impacted by the illnesses being researched as well as their cultural contexts. Herbal investing in toothpaste, for example, was a practice favored by women in Indonesia [18, 28]. The majority of participants were found to be older (55–75) than the desirable range of 31–45 for the continuance, growth, and sustainability of the herbal business [31, 32]. This is connected to the fact that young people overlook this sector and opt for white-collar careers; thus, there is a possibility that it may collapse in the near future. The majority of caregivers are illiterate since herbalists used productive school time to gain this information and expertise at the expense of schooling [20].

Interview results showed that the majority of herbalists were elderly males who were primarily uneducated. The cultural practices in the Elgon sub-region tend to exclude women. These narratives are best explained by age, gender, and dynamic knowledge. Socio-cultural and demographic traits such as gender, age, and literacy are all correlated with an individual's level of knowledge [33, 34]. Women are thus discouraged from working in this industry within the cultural framework of Elgon inhabitants, which explains their low participation rates. As a result, societal norms, habits, and levels of development have a significant role in motivating people to learn about using plants as medicine [34].

**Fig. 1:**
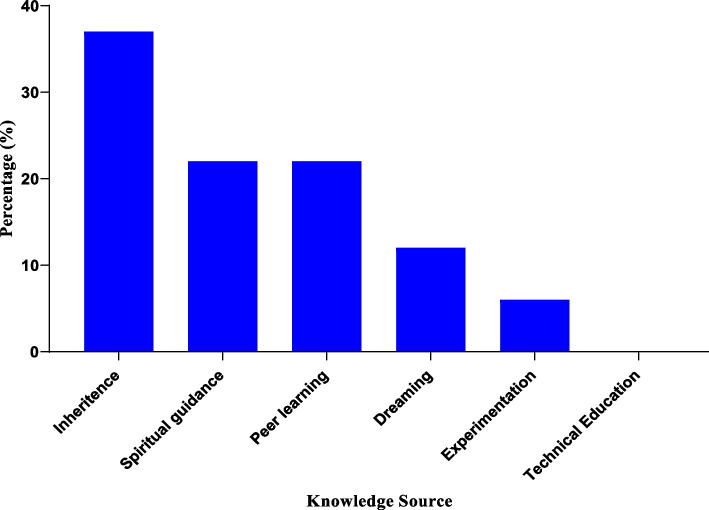
Source of knowledge of medicinal plants

More herbalists acquired their plant cancer treatment knowledge through inheritance (Fig. 1). Earlier studies have also shown that herbalists in Butelejja followed a similar path in acquiring herbal knowledge [18]. On the other hand, in Saudi Arabia, friends were the best resource for learning about plant therapy treatments. As a result, the variation is related to the cultural variations between the two research areas [35].

The most popular plant species used to treat cancer were *Tylosema fassoglensis* (Schweinf.) Torre & I and *Hydnora abyssinica. A. Br., Hydnora Africana, Rhoicissus tridentata* (L.f.) Wild & R.B. Drumm, *Azidarachata indica, Prunus Africana, and Kigelia Africana* (Lam). Benth*, Albizia coriaria,* and *Plectranthus cuanneus* (Table 2). These plant species are commonly used in this region because they are easily accessible, affordable, and, most importantly, are purported to be effective and safe for use by people [36]. Some plant species, like *Punus africana* (Hook. f) kalk and *Albizzia coriaria* Welw. ex oliv., were commonly used in cancer treatment in Kakamega County, Kenya [37]. Reviews conducted in Uganda and Kenya, in contrast, listed the following plant species: *Opunitia species*, *Daucus carota* L, *Cyperus alatus* (Nees) F. Muell, *Markharmia lutea* (Benth.) K. schum, *Oxalis corniculata* L, and *Catharanthus roseus* (L). G. Don, the *Spathodea campunulata* P. Beauv, ssp. nilotica (seem), *Microglossa pyrifolia* (Lam.) Kuntze, *Harungana madagascariensis, Cyphostema serpens* (A. Rich), *Aloe volkensii*, &Engl, *Toddalia asiatica*, *Annona muricata, Carica papaya*, *Molinga oleifera*, Entada abyssinica, Steud. Ex Rich, *Phyllanthus fischeri* Pax, *Sapium ellipticum*, *Shirakiopsis ellipticum (Hoscht)* Baill,*Fatumia sfricana* Benth, Ocimum *gratissimum,* and *Zanthoxylum paracanthum,* which were not cited in our study [15, 37, 38]. The growth of species is regulated by ecological variation and study time. For example, these systematic evaluations gathered plant species that had been employed for more than 10 years in various parts of Kenyan and Ugandan regions with a range of ecological and cultural conditions [13, 39].

**Fig. 2:**
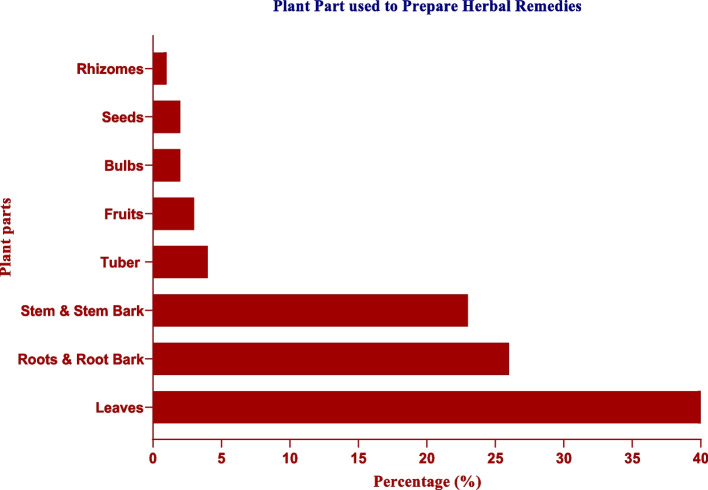
Plant Parts used to prepare herbal remedies

The most often used plant parts of cancer therapies were leaves (Fig. 2, Table 1). Because the removal of leaves does not harm the plant, they are readily available everywhere and are believed to contain high amounts of bioactive components [35, 39–42]. The inhabitants in the Berber region of Ethiopia and Palestine have been noted to have historically relied significantly on their distinct root, fruits, flowers and seeds cures. The difference is connected to the ailment under study (a human disease), the plant families and species in question, the ecological context, and the cultural context [43]. Elders' comments stressed the ease with which leaves can be prepared and the fact that they are more frequently seen, gathered, and thought to have medicinal properties than other plant parts, as reflected in their predominant use. These comments could be best appreciated in light of the resource availability hypothesis [34], which tends to contend "that the distribution of secondary chemistry within a given plant drives the selection of plant organs for medicinal purposes." The optimal defense theory offers a framework for comprehending why individuals might opt for roots from the same plant rather than leaves for medical purposes.

**Table 1:** Medicinal plants, their habits, cancer treated, and methods of preparation and route administration

Plant Family, Scientific Name & Voucher No	Local Name	Plant Habits	Plant Part(s) Used	Type of Cancer	Method of Preparation	Route of administration
Alliaceae, *Allium sativa*, -KA 006	Kachuum	Herb	Bulb	Cervical, colon, prostate & skin	Boiled & drunk (decoction); Burnt & applied on infected skin part	Oral and topical medication
Amaryllidaceae, *Allium sepa* L -KA 007	Kitungulu	Herb	Bulb	Stomach	Mixed with *Mangifera indica, aloe vera* & *Biden Pilosa,* boiled drunk (Concoction)	Oral
Apocynanceae, *Rouvoflavia vomitoria Afzel -*KA 008	Kitondwani	Shrub	Leaves, roots and stems	Colon & cervical cancer	Boiled & drunks (decoction)	Oral
Anarcardiaceae, *Mangifera indica* – KA 009	Kiyembe	Tree	Leaves	Prostate, cervical, breast colon, lung, GIT, skin intestinal uterine, esophageal bone & bone cancers	Boiled & drunk (decoction); Burnt & applied on infected skin part	Oral & topical medication
Asphodelaceae, *Aloe vera* – KA- 010	Kigadi	Herb	Leaves	Prostate, cervical, breast colon, lung, skin intestinal uterine, esophageal bone & bone cancers	Boiled and drunk (decoction) Burnt & applied on infected skin	Oral & topical medication
Asteraceae*, Biden Pilosa* L –KA 011	Kyikhamama (Tabululu)	Herb	Leaves, stem and roots	Prostate, cervical, breast colon, lung, GIT, skin intestinal uterine, esophageal bone & bone cancers	Boiled and drunk (decoction); Burnt & applied on infected skin	Oral & topical medication
Acanthaceae*, Amelia caurrhiza* Del – KA 012	Nandwasi	Herb	Leaves	Prostate, cervical, breast colon, lung, GIT, skin intestinal uterine, esophageal bone & bone cancers	Boiled and drunk (decoction); Burnt & applied on infected skin	Oral & topical medication
Asteraceae, *Conyza sumatrensis KA* 013	Namagoye	Herb	Leaves	Prostate, cervical, breast colon, lung, GIT, skin intestinal uterine, esophageal bone & bone cancers	Mixed *Dicrocephala integrifolia,* boiled & drunk (concoction) & burnt and applied on infected skin	Oral & topical medication
Asteraceae, *Dicrocephala integrifolia -*KA 014	Lunyabakana	Herb	Leaves	Prostate, cervical, breast colon, lung, GIT, skin intestinal uterine, esophageal bone & bone cancers	Mixed with *Vernonia adonsis* and *Mangifera indica, bolied* and drunk (concoction). Burnt & applied on infected skin	Oral & topical medication
Asteraceae, *Vernonia adonsis* Walp – KA 015	Kisola	Tree	Leaves	Prostate, cervical, breast colon, lung, GIT, skin intestinal uterine, esophageal bone & bone cancers	Mixed with *Kigelia Africana, Ananasi sativa* (Retz) Walker Boiled & drunk (concoction); Burnt and applied on infected skin	Oral & topical medication
Bignoniaceae*, Kigelia africana –* KA 016	Gufungo/Kifungo	Tree	Leaves, roots and stems	Prostate and breast cancer	Mixed with *Hydnora abyssinica* A and *Hydnora Africana,* boiled & drunk (Concoction); Burnt & applied on the infected skin	Oral & topical medication
Bromeliaceae, *Ananasi sativa* (Retz) Walker – KA 017	Nadanga		Leaves, roots and stems	Oesophageal cancer	Boiled and drunk (decoction) *Mixed with Erythrina abyssinica* Lam., and *Mormordica foetida*, boiled & drunk (concoction)	Oral
Celastraceae, *Maytnus senegalensis* -KA 018	Kikonje	Shrub	Leaves, roots and stems	Cervical cancer	Boiled and drunk (decoction) Mixed with *Rinus communis* and *Dioscaena fragrans,* boiled & and drunk (concoction)	Oral
Clusiaceae, *Garcinai Buchananii* Baker – KA 019	Kikameli	Tree	Leaves & stem	Prostate, cervical, breast colon, lung, GIT, skin intestinal uterine, esophageal bone & bone cancers	Boiled and drunk (decoction) Mixed with *Rinus communis* and *Dioscaena fragrans,* boiled *&* drunk (concoction)	Oral
Euphorbiaceae, *Rinus communis* L -KA 021	Mukakale	Herb	Leaves, roots and stems	Uterus cancer	Boiled and drunk (Decoction) Mixed with *Entada abyssinica* stued & *Mangfera indica* and boiled & drunk (concoction)	Oral
Dioscoreaceae, *Dioscaena bulbifera.* Linn -KA022	Liakunyu	Herb	Leaves & tubers	Cervical, breast & prostate cancers	Boiled and drunk (Decoction) Mixed with *Dioscaena fragrans* (L.) Ker-Gawl and *Cajanus Cajan*, boiled & drunk (concoction)	Oral
Draceananceae, *Dioscaena fragrans* (L.) Ker-Gawl—KA 023	Linzi	Shrub	Leaves	Cervical & colon	Boiled and drunk (decoction Mixed with *Hydnora africana* & *Hydnora abyssinica* A. Br and *Pseudarthria hookeri* Wight, boiled & drunk (concoction)	Oral
Hydnoraceae, *Hydnora africana* – KA 025	Mwoyogwemutaka	Herbs	Roots	Prostate & cervical cancer	Boiled & drunk (decoction) Mixed with *Tamarindus indica* and *Cajanus Cajan*, Boiled & drunk (concoction)	Oral
Fabaceae, *Cajanus Cajan—*KA 026	Zikolimbo	Herb	Leaves, stem & roots	Breast	Burnt and applied on infected skin	Topical medication
Fabaceae, *Erythrina abyssinica* Lam. Ex. DC – KA 027	Kitugulu	Tree	Stem & root bark	Cervical & oesophageal cancers	Eaten raw, Boiled & drunk (decoction Mixed with *Rumex usambarensis* & *Combretum mole* G-don and, drunk (concoction)	Oral & topical medication
Fabaceae, *Pseudarthria hookeri* Wight & Arn KA 028	Nakikofira	Herb	Leaves, stem & roots	Breast	Boiled and drunk (decoction), applied on infected skin	Oral & topical medication
Fabaceae, *Tamarindus indica* L -KA 032	Mukuwe	Tree	Fruit	GIT & prostate	Crushed and taken raw (infusion) or boiled & drunk (decoction)	Oral
Fabaceae, *Entada abyssinica* stued – KA 031	Kishembe	Herb	Leaves, stem & roots	GIT & prostate	Boiled and drunk (decoction)	Oral
Fabaceae, *Albizia coriaria* (Welwe.) ex Oliv KA 030	Kiluku	Tree	Root back	Prostate, cervical, breast colon, lung, GIT, skin intestinal uterine, esophageal bone & bone cancers	Boiled and drunk (decoction)	Oral
Lamiaceae*, Plectranthus cyanneus -*KA 033	Wobulaka (Tuliguuye)	Herb	Leaves, stem & roots	Skin cancer	Crushed and taken (infusion) or boiled and drunk (decoction)	Oral & topical medication
Lamiaceae, *Leonotis nepetifolia* (L) R -KA 034	Namusiriri	Herb	Leaves, stem & roots	Prostate, cervical, breast colon, lung, GIT, skin intestinal uterine, esophageal bone & bone cancers	Crushed and taken raw (infusion)	Oral
Lauraceae, *Persea Americana* -KA 035	Pekedo	Tree	Leaves & seeds	Skin cancer	Boiled and drunk (decoction)	Oral
Meliaceae, *Azedaracta indica* – KA 036	Murabaine	Tree	Leaves, roots and stems	Prostate, cervical, breast colon, lung, GIT, skin intestinal uterine, esophageal bone & bone cancers	Boiled and drunk (decoction)	Oral
Primulaceae, *Maesa lanceolata* Forrsk -KA 037	Kinywabanji	shrub	Leaves, roots and stems	Skin cancer	Boiled and drunk (decoction)	Oral
Rosaceae, *Prunus africana* (Hook.f). kalk -KA- 040	Gusasa (Kilumati)	Tree	Leaves, roots and stems	Prostate, cervical, breast colon, lung, GIT, skin intestinal uterine, esophageal bone & bone cancers	Mixed with *Ribia cordifera*. *Allophylus abyssinicus* P. Beauv, boiled & drunk (Concoction)	Oral
Myrtaceae, *Syzygium cumini* (L) Skeels- KA 038	Jambula	Tree	Leaves, roots and stems	Prostate, cervical, breast colon, lung, GIT, skin intestinal uterine, esophageal bone & bone cancers	Boiled and drunk (decoction) & burnt and applied on infected skin	Oral & topical medication
Polygonanceae, *Rumex usambarensis*—KA 039	Nankombi	Herb	Leaves, roots and stems	Prostate, cervical, breast colon, lung, GIT, skin intestinal uterine, esophageal bone & bone cancers	Boiled & drunk (decoction)	Oral
Rubiaceae, *Ribia cordifera*. Linn -KA 041	Kizambazambe	Herb	Leaves, roots and stems	Lung & skin cancer	Mixed with *Ribia cordifera*. & *Gouania longispicata* Engl. Linn, boiled & drunk (Concoction)	Oral
Sapindaceae, *Allophylus abyssinicus* P. Beauv – KA 043	Zipelele	Shrub	Leaves, roots and stems	Lung cancer	Boiled & drunk (decoction)	Oral
Rhmanceae, *Gouania longispicata* Engl. (Ait) -KA 042	Namayendeyende	Shrub	Leaves	Skin cancer	Boiled & drunk (decoction) & burn and applied on infected skin	Oral & topical medication
Simaroubaceae, *Harrisonia abyssinca* Olivia—KA 044	Netu (Nasambu Or Nefulo	Tree	Leaves, roots and stems	Prostate, cervical, breast colon, lung, GIT, skin intestinal uterine, esophageal bone & bone cancers	Mixed with *Gouania longispicat a* Engl. & *Lantana trifolia* boiled and drunk (concoction)	Oral
Verbenanceae, *Lantana trifolia*—KA 045	Namuserera	Shrub	Leaves, roots and stems	Cervical cancer	Boiled and drunk (decoction)	Oral
Urticaceae, *Urtica dioica* L – KA 046	Nettle	Herb	Root tuber	Skin cancer	Crushed & taken raw (infusion) or boiled & drunk (decoction	Oral
Vitaceae, *Cyphosteman adenocaule* (A. Rich -KA 047	Namakajo	Herb	Leaves & roots	Skin cancer	Mixed *Hypoxis hemerocallidea Fish* C. A. Mey. &Ave-Lall and *Psidium guajava* boiled and drunk (Concoction) and applied on infected skin	Oral & topical medication
Zingiberaceae, *Zingiber officinale -*KA 048	Tangawunzi	Herb	Rhizome	Oesophageal cancer	Eaten raw, Mixed with Hypoxidaceae. *Hypoxis* and *Rhoicisuss tridentata,* boiled & drunk *hemerocallidea Fish* C. A. Mey. &Ave-Lall (Concoction)	Oral
Vitaceae, *Rhoicisuss tridentata* (L.f) Wild & R.B Drumm- KA 051	Nakibondi	Shrub	Leaves & tubers	Prostate and breast	Crushed, and drunk (infusion) boiled& drunk (Decoction)	Oral
Hypoxidaceae. *Hypoxis hemerocallidea Fish* C. A. Mey. &Ave-Lall—KA 049	Mabondi Gemukyigona	Shrub	Tuber	Prostate, cervical, breast colon, lung, GIT, skin intestinal uterine, esophageal bone & bone cancers	Boiled and drunk (decoction) and applied on infected skin	Oral & topical medication
Myrtaceae, *Psidium* g*uajava* L. KA 050	Lipella	Tree	Leaves	Prostate, cervical, breast colon, lung, GIT, skin intestinal uterine, esophageal bone & bone cancers	Mixed with *Rhoicisuss tridentata* L.f) Wild & R.B & *Combretum mole* G-don Drumm boiled and drunk (Concoction)	Oral
Euphorbiaceae, *Margaritria discoidea* (Baill) Muell. Arg. -KA 052	Gulumati/Kilumati	Tree	Leaves and stem bark	Prostate, colon, cervical and breast	Boiled & drunk (decoction) and applied on infected skin (topical application)	Oral & topical medication
Sapindaceae, *Deinbollia* fulva -*fomentalla* Bak.f – KA053	Kifuti	Tree	Fruits, and leaves	Prostate, cervical, breast colon, lung, GIT, skin intestinal uterine, esophageal bone & bone cancers	Boiled & drunk (decoction) and applied on infected skin	Oral & topical medication
Phyllanthaceae, *Bridilia micrantha* (Hochst.) Bail-KA054	Kigakala	Tree	Leaves, roots and stems	Skin, prostate and cervical cancer	Boiled & drunk (decoction) and applied in infected skin	Oral & topical medication
*Combretaceae,* *Combretum mole* G-don- KA 055	Kimwanyimwayi	Shrub	Leaves	Skin, prostate and cervical cancer	Boiled & drunk (decoction) and (applied on infected skin	Oral & topical medication
Rubiaceae *Coffea arabica—*KA056	Emwanyi	Tree	Leaves and fruits	Prostate cancer and cervical cancer	*Squeezed and* the juice drunk (infusion) Mixed with *Mangifera indica* and *Deinbollia* fulva -*fomentalla* Bak.f boiled & drunk (Concoction) Fruit berries area rooted and added to tea	Oral

**Fig. 3:**
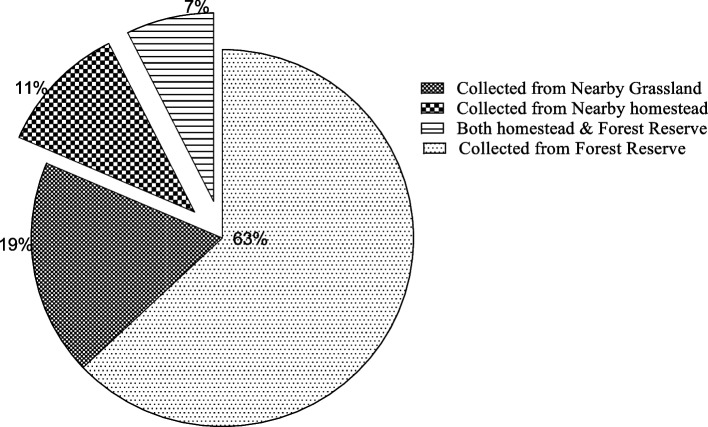
Source of medicinal plants

**Fig. 4:**
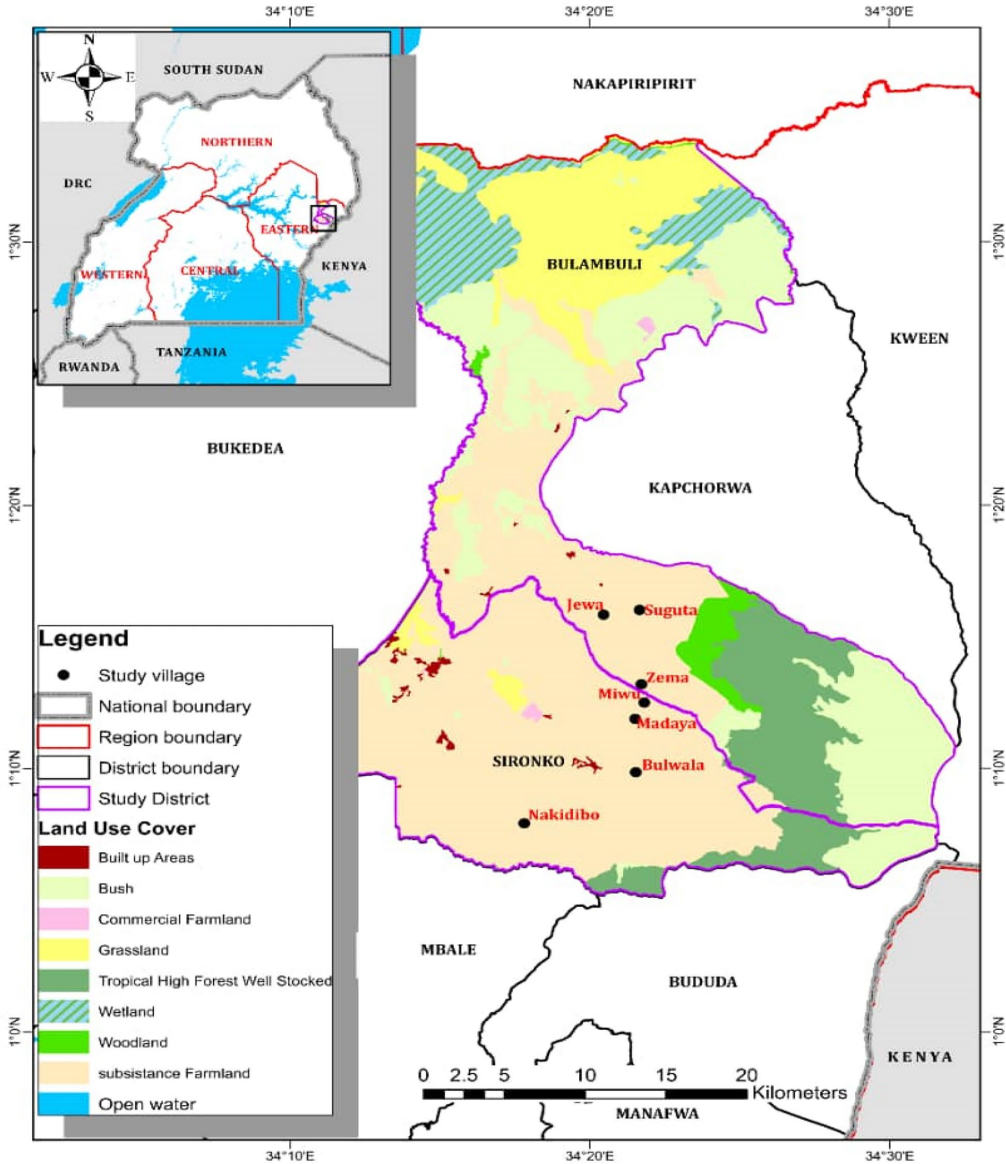
Map of Elgon Sub-region with Location of Sironko and Bulambuli districts

More plant species used in the preparation of herbal remedies were collected from the forest reserve (Fig. 3). Gathering medicinal practices is a common and long-standing activity in Uganda. It has been generally documented that the majority of plant species used to treat various illnesses are gathered from the wild in various locations around Ethiopia, Morocco, and Uganda [39, 41, 43, 44]. Result from Butelejja district of eastern part of Uganda revealed the reverse, which is related to the lack of a forest, making domestication and gathering from surrounding bushes as important sources of herbal medicine [18]. Elders in the Sironko and Bulambuli areas claimed that there was no need to domesticate herbal plants because the neighboring forest reserves already had a diversity of species (Fig. 4). In addition, the elders claimed that there was not enough land to support the production of both crops and herbal plants. In accordance with this, the "resource availability hypothesis" looks at the area where people collect plants and more broadly links local plant abundance or dominance with plant usage. This idea contends that the availability, accessibility, and intended use of plants in the wild are all driving factors to their domestication [40].

Findings showed that more plant species thought of therapeutic benefits against cancer belong to plant families Fabaceae and Asteraceae (Table 1). The predominance use of these families is due the fact that most plant species in these families are mainly herbs and shrubs, so grow quickly and available throughout the year [34, 40, 45]. Several parts of the world, notably Kenya [37], Ethiopia [45], Tanzania [46], Egypt, Iraq, Israel, Palestine, Jordan, Lebanon, Palestinian territories, Syria, and Turkey [29, 39, 42] are home to the majority of plant species from the Fabaceae and Asteraceae families. Apocynanceae, Bignoanceae, Moraceae, Rutaceae, Sapindaceae, Meliaceae, Caricaceae, Solananceae, and Malvaceae, on the other hand, were less prevalent in this region but more evident in a number of other regions of the world, including Sri Lanka and Morocco [43, 45, 47], Kenya [37], Ethiopia [45], Tanzania [46], Egypt, Iraq, Israel, Jordan, Lebanon, Palestinian territories, Syria, and Turkey [37, 48], and Lamiaceae [29], due to distinct in ecological and cultural diversity.

**Fig. 5:**
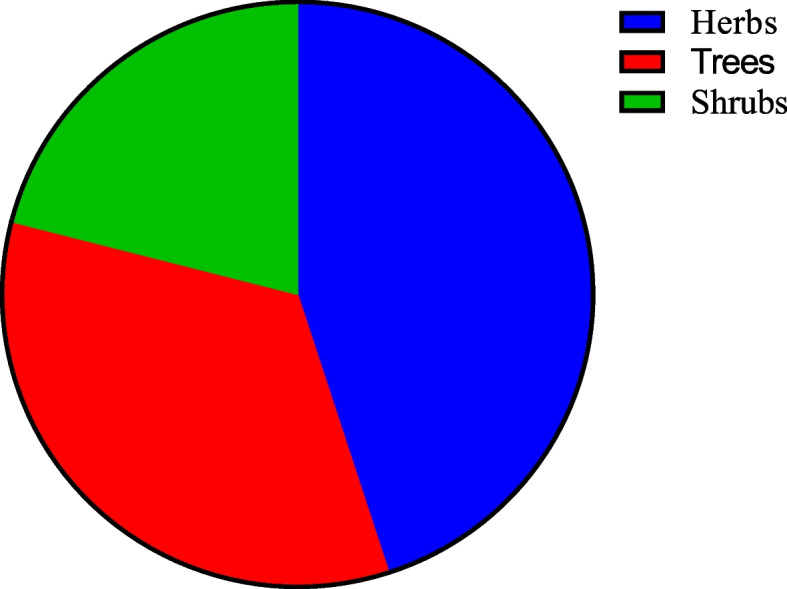
Plant habits

The results of the analysis revealed that more plant species utilized in cancer treatment were herbs and trees (Fig. 5). The use of herbs and trees to prepare herbal therapies has also been widely reported in Ethiopia and Morocco [44, 45]. This is connected to the fact that high quantities of flavonoids, alkaloids, phenols, tannins, and anthraquinones are thought to be responsible for these plants' use [34, 44, 49, 50].

**Fig. 6:**
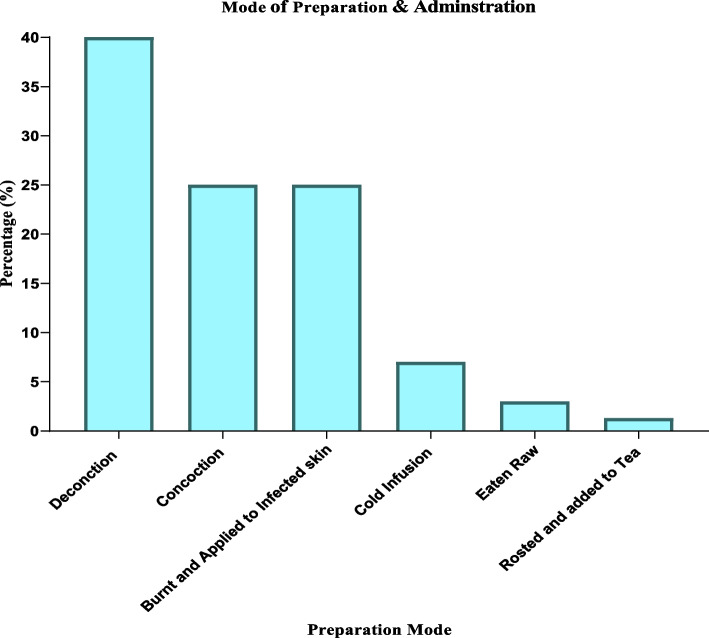
Modes of preparation & route of administration of herbal therapies

**Fig. 7:**
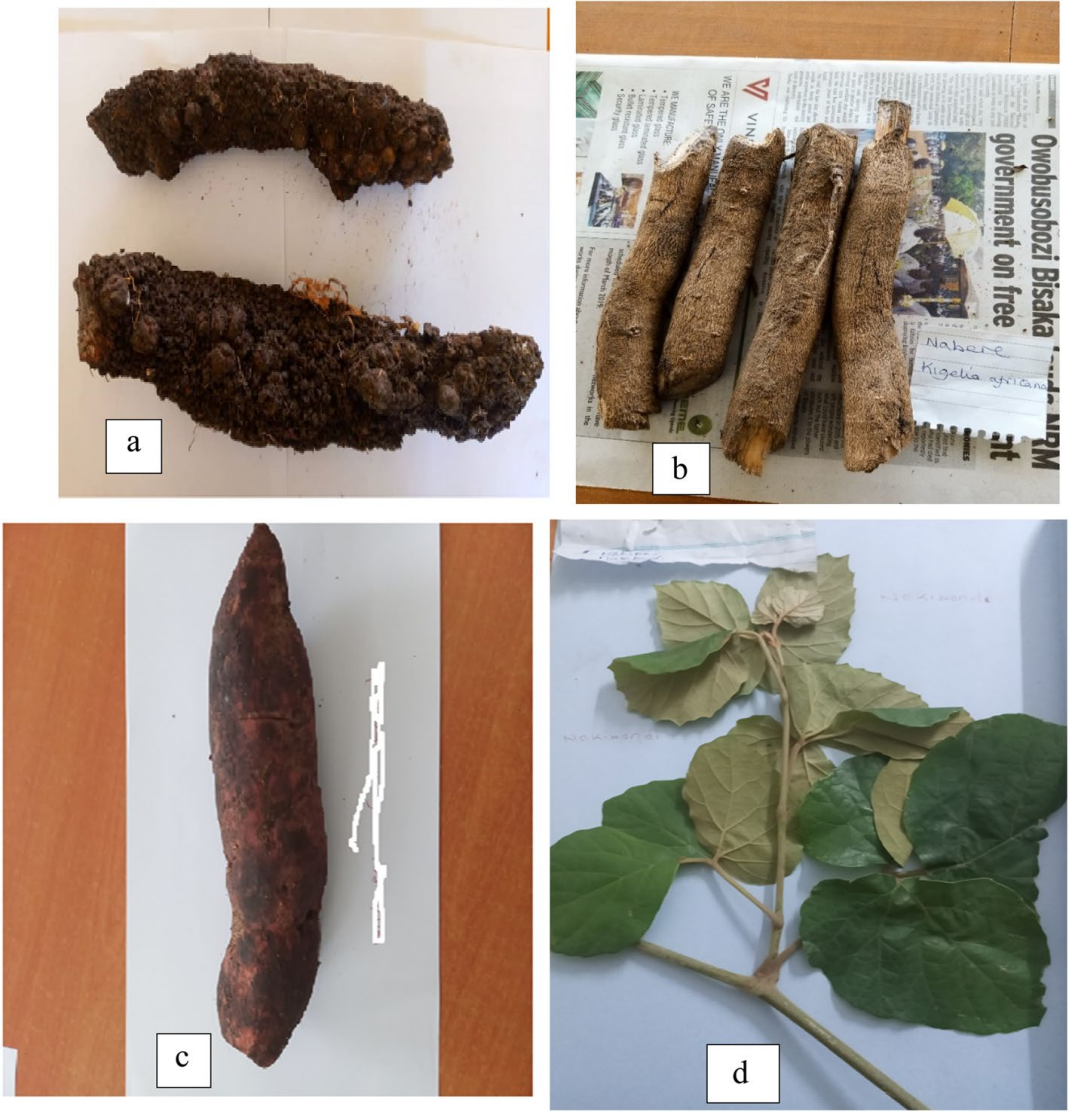
Plant Species used in the Treatment of Cancer in Sironko and Bulambuli in the Elgon Sub-Region. **a** Hydnora abyssinica. A. Br (Rhizomes) **b** *Combretummole G-don. ***c** Rhoicissus tridentata (L.f.) Wild & R.B. Drumm (Root tuber). **d** Rhoicissus tridentata (L.f.) Wild & R.B. Drumm (shoot)

More herbal medicines used to treat cancer were decocted and taken orally (Figs. 6 and 7). The decoction method is popular because it is thought to be the least expensive, easiest, and, most importantly, does not call for technical knowledge [12, 20, 43, 46]. Concoctions frequently reported to be employed in herbal preparation in Ethiopia, Kenya, and Gabon diverge from our study. This is explained by the widespread belief among herbalists that combining multiple plants gives more synergistic advantages in disease management than combining one plant (decoction) [13, 38, 42].

The plants used typically had a scored plant value above 50%, which denotes that they are preferred in the treatment of cancer (Table 2). Due to its versatility in treating multiple cancers in this area, *Tylossema fassoglensis* (Schweinf.) Torre& I scored the highest plant value (86%). *Tylossema fassoglensis* (Schweinf.) Torre & I is often used since it is thought to perform well either when decocted or concocted and has a higher assumed potential for curing several cancers. The phytochemical evidence revealed that *Tylossema fassoglensis* (Schweinf.) Torre &I harvested from the Elgon sub-region recorded the highest concentration of phenols, tannins, flavonoids, and alkaloids compared to those ever recorded in different locations of Uganda [38]. This species has been widely used to cure a number of ailments, including bacterial, viral, and fungal problems, despite the fact that it contains a higher concentration of chemical components [51].

**Table 2:** Preference ranking of medicinal plants

MPS	PPU	CT	KI (*n* = 10)										PV/100	PR
			A	B	C	D	E	F	H	I	J	K		
***Tylosema**** fassoglensis*(Schweinf.) Torre& I	Tubers	Prostate, cervical, breast colon, lung, skin, GIT intestinal uterine, esophageal bone & bone cancers	10	9	8	9	7	9	9	8	8	9	86	1^st^
*Hydnora abyssinica*.A. Br	Tuber	Prostate, cervical & breast cancer	9	8	7	8	8	8	7	7	7	7	84	2^nd^
*Hydnora africana*	Tuber	Prostate, cervical, & breast cancer	8	8	8	8	8	9	9	7	8	7	80	3^rd^
*Rhoicissus tridentata* (L.f.) Wild & R.B. Drumm	Leaves, stem and tuber	Prostate, Colon & GIT cancers	6	7	8	7	8	7	7	7	7	7	78	4^th^
***Albizia coriaria*** (Welwe.) ex. Oliv	Tuber	Skin & colon cancer	8	8	8	8	8	7	7	8	7	7	76	5^th^
***Azadirachta indica***	^7^	Lung cancer	8	8	8	7	7	8	8	6	7	7	74	6^th^
***Prunus africana (*(Hook.f) kalk**	Bark and leaves	Prostate & cervical,	6	7	8	7	7	8	6	7	7	7	72	7^th^
*Kigelia Africana*	Fruit	Prostate & esophageal,	7	7	6	8	7	7	7	6	7	7	69	8^th^
***Syzygium cumini* (L)Skeel**	Leaves	Colon & GIT cancers	7	7	7	6	6	7	6	5	7	7	65	9^th^
*Erythrina abyssinica Lam*. Ex. Dc	Stem & root barks	Colon	5	5	6	6	5	6	6	7	7	7	60	10^th^

Where *MPS* Medicinal Plant Species, *PPU* Plant Part Used, *KI* key informant, *PV* Plant Value and, *PR* Plant Rank &, *CT* Cancer type)

With no exception, the informant consensus factor (ICF) for all cancers listed in this study was high (0.84–0.95) (Table 3). The high ICF recorded signified that the herbalists had grasped a clear understanding of the plant used in cancer treatment, and there was well-designed communication among the herbalists. A low ICF (0.0619) was observed by [18] and was ascribed to the misinterpretation of cancer with other illnesses since it shares symptoms such as headache, nausea, vomiting, and diarrhea. More crucial, however, is the dearth of competent cancer diagnostic facilities in rural locations.

**Table 3:** Informant Consensus Factor (ICF)

Cancer Type	No. of use citation N_ur_	No. of species N_taxa_	ICF
Prostate cancer	224	37	0.84
Cervical cancer,	222	35	0.85
Breast cancer	208	21	0.90
Colon cancer	211	24	0.89
Lung cancer	201	14	0.94
Skin cancer	203	16	0.93
Intestinal cancer	198	11	0.95
Uterine cancer	199	12	0.94
Esophageal cancer	201	14	0.94
Bone cancer	198	11	0.94
Gastrointestinal cancers	200	13	0.94

The current investigation showed that all plants obtained a high reliability level above 0.5 (Table 4). While *Hydnora abyssinica* rated the highest (93.9%) for treating prostate cancer*, Tylosema fassoglensis* got the highest fidelity level (88.1%) for treating numerous cancers. This showed that the herbs used in this study were typically quite useful for treating cancer*.* This is consistent with the level of fidelity (90%–100%) observed in the Hidaya zone of southern Ethiopia and Jeju Island of Korea with regard to the use of medicinal plants to treat a variety of ailments. This high level of loyalty demonstrated the importance and trust that communities all across the world have in herbal remedies [13, 52, 53]. The high trustworthiness of the plants obtained from these species is partially attributed to the high phytochemical component of the concentration of tannin, alkaloids, polyphenols, and flavonoids [39].

**Table 4:** Fidelity Level (FL) of most commonly used plants in cancer by key informants

Plant species	Therapeutic use	Lp	Lu	FL (%)
*Tylosema fassoglensis* (Schweinf.) Torre& I	Prostate, cervical, breast colon, lung, skin intestinal uterine, esophageal bone & bone cancers	32	36	88.9
*Hydnora abyssinica A. Br*	Prostate cancer	31	33	93.9
*Hydnora africana*	Prostate	28	33	84.8
*Rhoicissus tridentata* (L.f.) Wild & R.B Drumm	Prostate and Colon	35	39	89.7
*Azidarachata indica*	Lung cancer	35	38	92.1
*Prunus africana* (Hook.f.) Kalk	Prostate	28	34	82.4
*Kigelia africana*	Prostate & oesopahagel cancer	27	34	79.4
*Albizia coriaria* (Welw) ex. oliv	Skin cancer	35	40	87.5
*Syzygium cumini* (L) Skeels	Gastrointestinal cancer	12	16	75.0
*Plectranthus cuanneus*	Gastrointestinal cancer	05	07	71.4

The herbalists said that some plants have been trusted and proved beneficial to several patients with different cancer conditions. In every herbal therapy preparation, at least parts of the plant are included in the concoction or may be used alone. These responses are best understood by applying the "Plant Values Hypothesis," which proposes that "the use of plants by a given community as medicine, food, or construction is directly related to the botanical family, life form, and local abundance and affordability."

## Conclusions and recommendations

The ethnobotanical survey in Sironko and Bulambuli districts revealed the over-reliance of several plant species to treat different cancers due to limited conventional options and their several shortcomings. The majority of medicinal plant species used belonged to the Fabaceae and Asteraceae families and were mainly herbs and trees. Leaves were more commonly used to prepare herbal therapies from decoction and orally administered. The main method for transferring herbal knowledge down the generations was inheritance. The most widely preferred and trusted plants in cancer treatment were *Tylosema fassoglensis* and *Hydnora abyssinica*. A. Br., *Hydnora africana, Rhoicissus tridentata* (L.f.) Wild & R.B. Drumm, *Albizia coriaria*, *Azidarachata indica, Prunus africana, Kigelia africana*, *Syzygium cumini* (L.) Skeels, and *Plectranthus cuanneus*. More research should be done on phytochemistry, toxicity, and in-vitro and in-vivo efficacy trials. This may increase the potential for the pharmaceutical sector to produce newer and more potent anticancer drugs.

### Supplementary Information

**Additional file 1.**

## Data Availability

All data generated or analyzed during this study are included in this published article.
