# Supplementary material for: Medicinal plants used in the management of cancers by residents in the Elgon Sub-Region, Uganda
Source: BMC Complement Med Ther. 2023 Dec 12;23:450. doi: 10.1186/s12906-023-04273-5 (PMC10714536; doi:10.1186/s12906-023-04273-5)
Supplement: Supplementary file 1 — Additional file 1. [file 12906_2023_4273_MOESM1_ESM.pdf]

QUESTIONNAIRES TO THE RESPONDENTS

Dear participants

I am **Ali Kudamba**, a Ph.D. student at Makerere University, and I am working on a study on the medicinal plants utilized in the Elgon subregion of Uganda to treat cancer. I humbly ask that you take part in this research. You are free to participate or not in this study at your discretion, and you are free to leave the study at any time. Your responses will be used exclusively for research and will be kept in strictest confidence. In light of this, none of our team member should ever ask you to disclose or write your identity down. Please feel free to answer the questions in the manner you see fit if you have agreed to participate in this study. All responses given in this respect are accurate, and the researcher will record each one as it is delivered.

PART ONE
SOCIO-DEMOGRAPHIC DATA

1. Gender

a) Male ☐ Female ☐

b) Age bracket of the respondents

25- 30 ☐ 31 -35 ☐ 36-40 ☐ 41- 45 ☐ 46-50 ☐

51- 55 ☐ 56-60 ☐ 60 above ☐

c) i) Nationality Ugandan ☐ Non- Ugandan ☐

ii) If non-Ugandan specify your nationality

....

……………………………………………………………………………………….

c) Marital Status

Single ☐ Married ☐ Divorced ☐ widowed: ☐

d) Education status

None ☐ primary level ☐ secondary ☐ tertiary & university): ☐

e) Source of income

peasant ☐ civil service ☐ Business ☐ other specify

………………………………………………………………………………………

f) Experience in the herbal sector: 10- 15 years ☐ 16 -20 years ☐

above 21 ☐

g) Source of herbal medicine knowledge:

District………………Sub-county…………………………Village ……………......

PART TWO

DIAGNOSIS & TREATMENT

2. a) i) Do we have a cancer patient in this area?

Yes ☐ ☐

ii) If yes how do you know that they are suffering from cancer:

Clinical records ☐ Signs and symptoms ☐

iii) If signs and symptoms what are the common cancers

....

....

....

....

b) What are the most common cancer types of cancer in this area

Prostate cancer ☐ Breast cancer ☐ Cervical cancer ☐

Stomach cancer ☐ Colon Cancer ☐ Oesophageal cancer ☐

Uterine fibroid ☐ GIT cancer ☐ Lung cancer ☐

c) Other types of cancer specify

....

....

....

d) What are the signs and symptoms of prostate cancer

....

....

....

3. a) What options do patients use to treat cancer in this area

Medicinal plant only ☐ Medicinal plants and counseling: ☐

Medicinal plants & spiritual processes ☐

Medicinal plants and conventional drugs ☐

b) Other treatment options

………………………………………………………………………………………

………………………………………………………………………………………

………………………………………………………………………………………

………………………………………………………………………………………

4. Do you use plants to treat all types of cancer?

Yes ☐ No ☐

6. Approximately, how many medicinal plants do you know to treat cancer

…………………………………………………………………………………………

…………………………………………………………………………………………

1. How long do you take for cancer patients to heal?
   One month ………………………………………………………………………..
   Two months ……………………………………………………………………….
   Three months ………………………………………………………………………
   Four months and above …………………………………………………………….

PERCEPTIONS AND OPINIONS ON CANCER PREVENTION

2. What advice can you give to people to prevent cancer disease
   ………………………………………………………………………………………

   ………………………………………………………………………………………

   ………………………………………………………………………………………
3. Do you think medicinal plants can effectively treat cancer?

   ………………………………………………………………………………………

   ………………………………………………………………………………………

   ………………………………………………………………………………………

4. Do you think modern medicine can also treat cancer effectively?

....

....

....

5. Why do think a person should choose traditional medicine instead of modern medicine for cancer treatment (Please write in the respondents' exact words)

....

....

....

....

PART THREE

COMMON PLANTS ARE USED FOR CANCER TREATMENT PLUS OTHER DISEASES.

**Local Name (Dialect)**	**Family**	**Scientific Name**	**Plant Habit**	**Part**	**Cancer type and other disease**	**Mode of preparation & administration**
					Cancer type<br><br>Others	
					Cancer type<br><br>Others	
					Cancer type<br><br>Others	
					Cancer type<br><br>Others	
					Cancer type<br><br>Others	
					Cancer type<br><br>Others	
					Cancer type<br><br>Others	

Name of researcher …………………………………..........… Sign …………………………

GOOD LUCK- GOD BLESS YOU
